# Potential new tool for anemia screening: An evaluation of the performance and usability of the TrueHb Hemometer

**DOI:** 10.1371/journal.pone.0230333

**Published:** 2020-03-12

**Authors:** Megan Parker, Kelsey Barrett, Maria Kahn, Dominira Saul, Pooja Bansil, Charlotte Tawiah, Nicole Advani, Stephanie Zobrist, Tala de los Santos, Emily Gerth-Guyette

**Affiliations:** 1 PATH, Seattle, Washington, United States of America; 2 Akendi, Ottawa, Ontario, Canada; 3 Kintampo Health Research Centre, Kintampo, Brong-Ahafo Region, Ghana; Pennsylvania State University, UNITED STATES

## Abstract

In low- and middle-income countries, many women experience anemia during pregnancy due to insufficient dietary intake of key micronutrients, parasitic infections, hemoglobinopathies, and chronic infections. Maternal anemia increases perinatal risks for both mothers and infants, and slow progress to reduce the prevalence may be due in part to the lack of affordable tools to quantify hemoglobin levels in antenatal care (ANC) clinics. A simple, inexpensive, accurate, and robust diagnostic is needed to measure hemoglobin in ANC. This study evaluated the performance and usability of the TrueHb Hemometer. A cross-sectional diagnostic accuracy study was conducted to compare the accuracy of the TrueHb and the HemoCue® 201+ using capillary samples. Next, analytical performance (precision, coefficient of variation, R^2^) of the TrueHb was evaluated in varying environmental conditions using linearity panels with serial dilutions of venous blood samples. Lastly, the usability of the TrueHb Hemometer was assessed across three domains (effectiveness, efficiency, and satisfaction) by 20 ANC providers in Ghana. Capillary blood test results were not well correlated (R^2^ = 0.35) between the TrueHB and HemoCue201+, but 80% of TrueHb measurements were within +/-1.0 g/dl of the HemoCue® 201+ hemoglobin values. Precision tests indicated similar mean values across the three environmental conditions (CV<6%). At 21°C, the TrueHb follows a linear relationship (R^2^≥0.96) but does not generate accurate readings below 4.0 g/dl. At 30°C and 37°C, the TrueHb follows a linear relationship (R^2^ > 0.90) but begins to underestimate the hemoglobin concentration below 7.0 g/dl. The usability study identified potential failure modes due to inadequate instructions and device feedback. With some modifications, both to the product and to the instructions for use, the TrueHb may be suitable for use in ANC settings to help fill the diagnostic gap for anemia screening during pregnancy. Further testing is required with anemic populations in LMIC settings.

## Introduction

Over one-third (38 percent) of pregnant women experience anemia during the gestational period, and approximately 800,000 are clinically diagnosed as having severe anemia.[1, 2] During pregnancy, mothers have increased requirements of energy, protein, vitamins, and minerals to meet maternal needs and infant growth demands. Although anemia can result from multiple underlying factors, in low- and middle-income countries (LMICs), most women experience anemia during pregnancy due to insufficient dietary intake of key micronutrients, such as iron, folic acid, and vitamin A; parasitic infections, including malaria, hookworm, and schistosomiasis; hemoglobinopathies; and chronic infections, such as HIV and tuberculosis.[2]

Maternal anemia is problematic in that it increases perinatal risks for both mothers and infants such as premature labor,[3,4,5,6,7] gestational diabetes,[4] preeclampsia,[4] acute heart failure,[4] postpartum hemorrhage,[4,8] severe postpartum infection,[3,4] and low birthweight or small for gestational age infants,[3,9,6,10,7] as well as increased morbidity and mortality [4, 11,12, 8–13]. For infants, many of these outcomes can contribute to impaired growth and cognitive development, with the potential for lifelong impact. To limit this intergenerational cycle of malnutrition, global and national recommendations call for interventions during pregnancy to reduce maternal anemia (e.g., iron and folic acid supplements) and improve health outcomes of infants and their mothers [2].

Over the past two decades, little progress has been made to reduce the prevalence of maternal anemia in LMICs.[14,15] In 2012, the World Health Assembly committed to achieving a 50 percent reduction of anemia in women of reproductive age (15 to 49 years old) by 2025. The World Health Assembly has called upon nations to review their health policies, infrastructure, and resources and implement strategies to prevent and control anemia.[14] In countries where the prevalence of anemia is at least 40 percent among pregnant women, the current World Health Organization (WHO) antenatal care (ANC) guidelines recommend testing women’s hemoglobin status at 12, 26, and 36 weeks of pregnancy and providing daily iron and folic acid (IFA) supplements (30 mg to 60 mg of elemental iron and 400 mg folic acid) from 12 to 40 weeks of pregnancy.[2] Accordingly, anemia screening is important both at the individual and population-levels, to inform patient care and treatment, as well as to guide public health interventions and national policies.

One reason for stagnated progress in reducing maternal anemia may be due to the lack of adequate tools to measure hemoglobin in places where women receive ANC. ANC facilities may not have the capacity or infrastructure to use laboratory methods such as hematology analyzers and currently available point-of-care (POC) tools may be too costly.[2,16] Currently, the HemoCue® 201+ (HemoCue AB, Ängelholm, Sweden) portable hemoglobinometer is the POC standard reference diagnostic for measuring hemoglobin concentration in capillary blood, particularly in settings where automated hematology analyzers are not affordable, available, or feasible for use. However, the cost of the HemoCue® may limit its application in routine ANC. As a result, many ANC facilities do not have access to POC hemoglobinometers. They may rely on other methods, such as the WHO color scale or physical examination of clinical pallor (e.g., conjunctiva, capillary refill in hands) to determine severe anemia (Hb<7.0 g/dl). While these methods have their merits, a quantitative tool is preferable, as it is less subjective and better able to produce accurate results and therefore inform treatment options.[17,18]

In places that lack the tools or capacity to conduct any anemia screening, ANC workers may distribute IFA supplements to all women, regardless of their anemia status. This blanket distribution strategy fails to stratify women according to their risk of anemia and may contribute to suboptimal rates of compliance with the IFA treatment. Women may not be motivated to take IFA supplements in the absence of a clear clinical diagnosis based on an individual hemoglobin measurement, particularly given the potentially unpleasant side effects of the treatment regimen.[19]

In order to align practices with the WHO ANC guidelines, a simple, inexpensive, accurate, and robust diagnostic is needed to measure hemoglobin in ANC in LMICs. The optimal device must be appropriate with respect to device cost, usability among target end users, acceptance among pregnant women, and sufficient accuracy for clinical decision-making.[20] In addition to the HemoCue, several other portable hemoglobinometers have been developed and released in recent years.[21] The TrueHb Hemometer System (Wrig Nanosystems, New Delhi, India), a new hemoglobinometer, represents one product that may fill the diagnostic gap for hemoglobin measurement in ANC.

Limited data is available on the performance of the TrueHb, particularly using capillary blood samples.[22,23] Two studies have investigated the diagnostic accuracy of the TrueHb using venous samples. Neogi and coworkers (2016) reported the TrueHb to perform better with venous than capillary blood samples for most parameters: sensitivity (74% vs 82%), specificity (87% vs 78%), PPV (86% vs 81%), NPV (75% vs 80%), ROC (0.81 vs 0.80), and correlation (r = 0.81 vs 0.77) to a hematological autoanalyzer.[22] Another study examined TrueHb’s performance using venous samples against an automated hematology analyzer (Sysmex XI 1800i) and found the two devices were strongly correlated (r = 0.99), with only a small bias (-0.02), and good precision (CV 2.22%).[23] Only one study has examined the diagnostic accuracy of the TrueHb using capillary samples against a hematological autoanalyzer, for anemia screening. In this study, 53% of the participants were anemic and 12% had severe anemia (mean (SD): 11.8(2.8) g/dl; range 1.3–22.0 g/dl]. The TrueHb had sensitivity and specificity values of 82% and 78%, respectively, and was well correlated with the autoanalyzer (r = 0.77).[22]

These data, along with early estimates of the test cost suggest that the TrueHb is a promising new tool for this use case. In addition to adequate performance, any new anemia screening tool must be able to be used by ANC providers. For the purposes of this evaluation, PATH took a multidisciplinary approach to both externally validate the performance of the diagnostic as well as better understand how it could be used in target settings by target users. The overall aim of this assessment was to evaluate both the usability and the performance of the TrueHb Hemometer in the context of filling the diagnostic gap for maternal anemia screening.

## Methods

A three-part evaluation of the TrueHb Hemometer was conducted. Verification testing was two-part. First the diagnostic accuracy of the TrueHb Hemometer was compared to the reference standard, HemoCue® 201+, using capillary blood samples. Second, we investigated the analytical performance of the TrueHb using linearity panels contrived through serial dilution of venous blood samples. Lastly, we conducted a usability evaluation of the TrueHb Hemometer with antenatal care providers in rural Ghana in order to assess operational fit and inform product introduction in antenatal care settings.

### Ethics

Ethical approval for the capillary study was obtained from the PATH Research Ethics Committee in April 2018. The protocol for the venous study and usability studies were reviewed by the Research Determination Committee in April 2018 and received a non-human-subjects research determination ethical approval. The protocol for the usability study was also submitted to the Kintampo Health Research Centre Institutional Ethics Committee and given full ethics approval. Written consent was obtained from all participants in the capillary study and the usability study.

### Capillary study

#### Study design

We conducted a cross-sectional study with 50 healthy volunteers to assess the performance of the TrueHb using fresh capillary blood samples. The Hemocue 201+ was used as the reference standard. The study team recruited and consented volunteers from the PATH office in Seattle, WA, USA, between May 21 and May 30, 2018.

#### Participants

Participants were at least 18 years of age and a PATH-Seattle staff member. The study excluded participants who self-reported as pregnant, receiving a blood transfusion within the last two months, or were ill within the past two weeks. For each participant, we collected the volunteer’s gender, age, and hemoglobin test results.

#### Blood collection

Each volunteer underwent two finger pricks and donated two fresh capillary blood samples, one for use with the TrueHb Hemometer and the other for use with the HemoCue ® 201+ device. Study staff drew blood from the participant’s right and left middle fingers, respectively. The first two blood drops were wiped away and the third drop of blood was applied to the test. If insufficient blood was drawn with the first finger prick, a second finger prick was done using the ring finger.

#### Analysis

Descriptive statistics were calculated (mean, SD, range) for each of the two devices. To assess agreement, linear regression was used to evaluate the correlation between the hemoglobin values produced by the TrueHb and the HemoCue® 201+ tests. To further evaluate the degree of agreement, the difference between the two POC devices was plotted against the mean value of the two POC devices in a Bland-Altman graph. As clinically acceptable limits of agreements for hemoglobin are within +1.0 g/dl (based on a 6% estimate for allowable method bias), test performance was calculated by determining the percent of TrueHb results that fell within 1.0 g/dl of the HemoCue® 201+ results. All statistical analyses were performed using Stata 13.0 (Statacorp, College Station, TX).

#### Venous study

*Study design*. To evaluate the analytical performance of the TrueHb in measuring hemoglobin across the dynamic range, we purchased de-identified, venous blood sample from Plasma Labs International (Everett, WA). Three venous samples were used to make three unique dilution series. Each sample was spun down and serially diluted using the same plasma generating 11 different concentrations. Precision testing was done in five replicates, at 21°C and 24% humidity, 30°C and 50% humidity, and 21°C and 33% humidity. Linearity was evaluated in four replicates with three donors at 21°C and 32% RH, two donors at 30°C and 56% RH, and two donors at 37°C and 50% RH. TrueHb precision and linearity were assessed using established performance criteria of ±1.0 g/ dL Hb (6% CV) from the HemoCue® reference test result.

#### Analysis

To determine precision, descriptive statistics were calculated (mean, SD, CV%) for each donor using the TrueHb in three different conditions. For linearity, TrueHb replicates were plotted and a linear fit model was assessed. All statistical analyses were performed using Stata 13.0 (Statacorp, College Station, TX).

### Usability study

#### Study design

To assess the usability of the TrueHb, we used a methodology based on ISO 9241–210 human-centered design principles, which measures usability across three domains: (1) effectiveness, (2) efficiency, and (3) satisfaction. Seven rural health clinics in the Brong-Ahafo Region of Ghana were selected as study sites for the usability testing. These sites were selected purposively due to their remote location and daily provision of ANC services and were staffed by ANC providers including midwives and nurses.

#### Participants

Participants were purposively sampled based on their job title, with the goal of enrolling approximately three participants per facility until reaching our total sample size of 20. Twenty participants is a valid standard to achieve saturation for a usability study of this type, which was exploratory, ethnographic, and focused on problem discovery.[24]

#### Process

During each usability testing session, the participant was asked to read the instruction booklet, watch a training video, and complete four tasks using the device while voicing his or her thought process aloud: (1) measuring a “patient’s” hemoglobin, (2) setting the batch code, (3) accessing a specific result stored in the device’s memory, and (4) taking another measure of a “patient’s” Hb. The study team did not provide any aid initially. If participants struggled with a task, the study team provided hints to help users through the process. Synthetic blood was used in place of actual blood, allowing participants to manipulate a sample of similar viscosity and color. These tasks represent common usage scenarios expected of device users. Tasks were followed by qualitative self-assessments and self-report ratings, in addition to four qualitative post-task probes. The sessions concluded with post-test questions, including interpreting results and providing information on education, work history, and routine practices for anemia screening.

#### Analysis

For each task, metrics on task success (pass, fail, pass with hints), total task time, and ease of use rating (five-point Likert scale) were collected. Total task time is an indicator of user efficiency. Shorter task times on subsequent identical tasks are indicative of learnability. For the analysis of common errors, we used a severity scale based on three categories: (1) catastrophic, (2) critical, and (3) major. Catastrophic issues are those that should be fixed before product use as they cause false results and do not alert the user that the result is invalid. Critical issues are those that should be fixed immediately, as they prevent the user from completing a task. Major issues are those that slow down task completion and increase the risk of human error during the task.

## Results

### Capillary study

**Fig 1:**
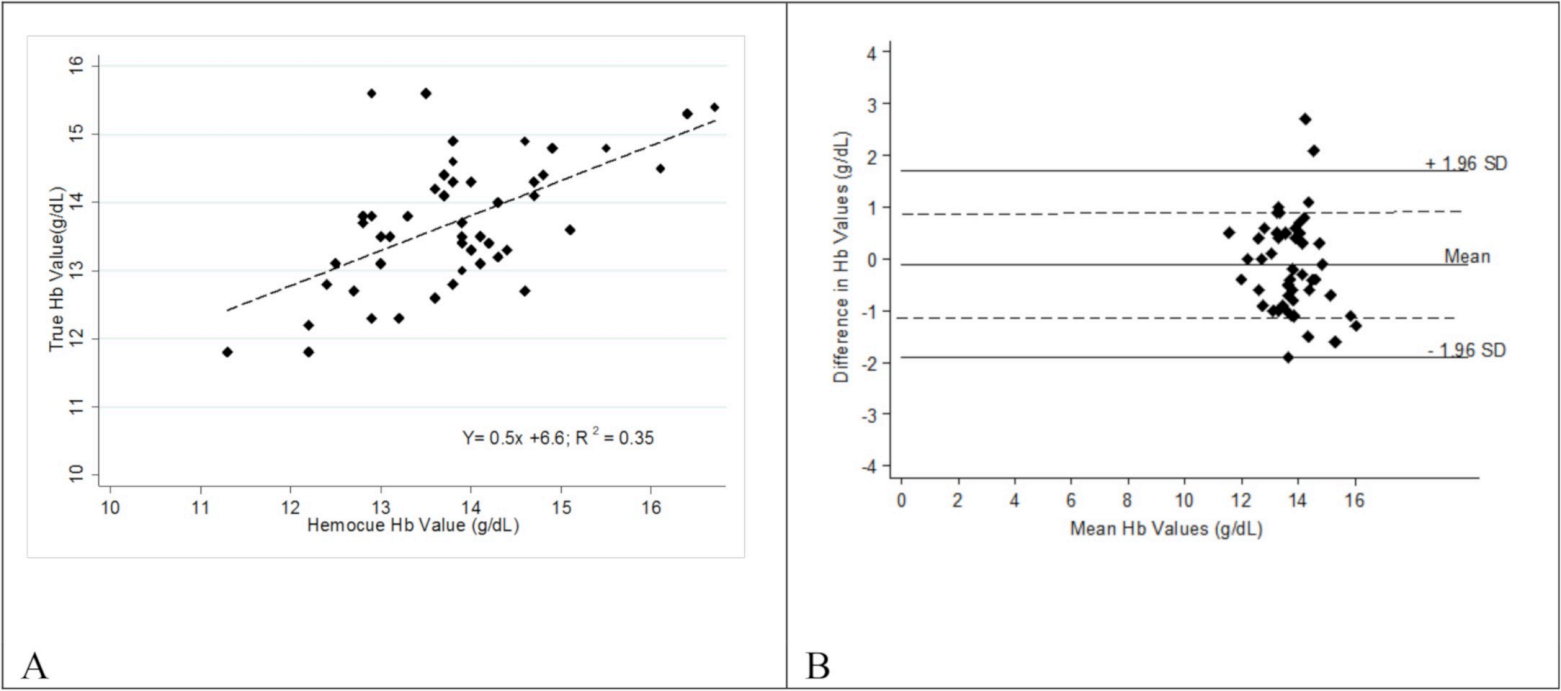
A. The correlation between the TrueHb and HemoCue® 201+ hemoglobin readings. B. A Bland Altman plot illustrating the bias (difference) between the TrueHb and HemoCue® 201+ hemoglobin readings, plotted against the average of the TrueHb and HemoCue® 201+ hemoglobin values.

Capillary blood samples were obtained from 50 participants (43 females and 7 males). The mean (sd) hemoglobin values produced by the TrueHb were 13.7 (0.9) g/dl with a range of 11.8–15.6 g/dl. The mean (sd) hemoglobin values produced by the HemoCue® 201+ were 13.8 (1.1) g/dl with a range of 11.3–16.7 g/dl. The correlation between the two POC tests were plotted (Fig 1A). Best fit shows R2 = 0.35. The Bland Altman shows 80% of the TrueHb measurements were within +/-1.0 g/dl of the HemoCue® 201+ hemoglobin values (Fig 1B).

### Venous study

Table 1 shows precision of the TrueHb diagnostic using three donor blood samples at three different temperature and humidity conditions. For each donor, the mean values were similar across 21°C and 24% humidity and 30°C and 50% humidity, and the coefficient of variation (CV) (%) were below 6% (Table 1).

**Table 1:** Precision of the TrueHb diagnostic in three donor blood samples at three different temperature and humidity conditions.

	Donor 1		Donor 2		Donor 3	
	Mean (SD); Range	CV (%)	Mean (SD); Range	CV (%)	Mean (SD); Range	CV (%)
**21⁰C; 24% Humidity**						
**True Hb (g/dL)**	11.9 (0.5) 11.0–12.3	4.5	13.1 (0.3) 12.8–13.7	2.6	15.2 (0.4) 14.5–15.6	2.8
**21⁰C; 33% Humidity**						
**True Hb (g/dL)**	11.7 (0.2) 11.4–12.0	1.9	13.3 (0.1) 13.2–13.4	0.8	14.9 (0.6) 14.0–15.5	3.9
**30⁰C; 50% Humidity**						
**True Hb (g/dL)**	12.1 (0.5) 11.2–12.5	4.4	13.7 (0.5) 13.3–14.4	3.3	15.4 (0.5) 14.6–15.8	3.2

SD: Standard Deviation

CV: Coefficient of Variation

**Fig 2:**
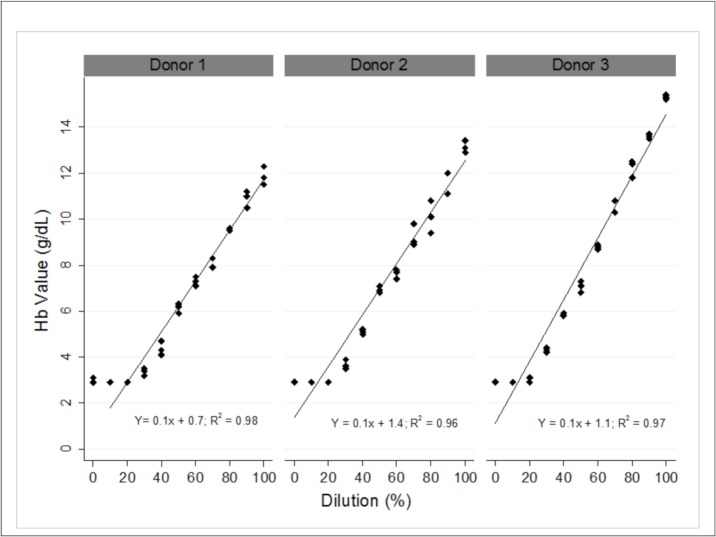
An assessment of TrueHb linearity at room temperature (21°C).

**Fig 3:**
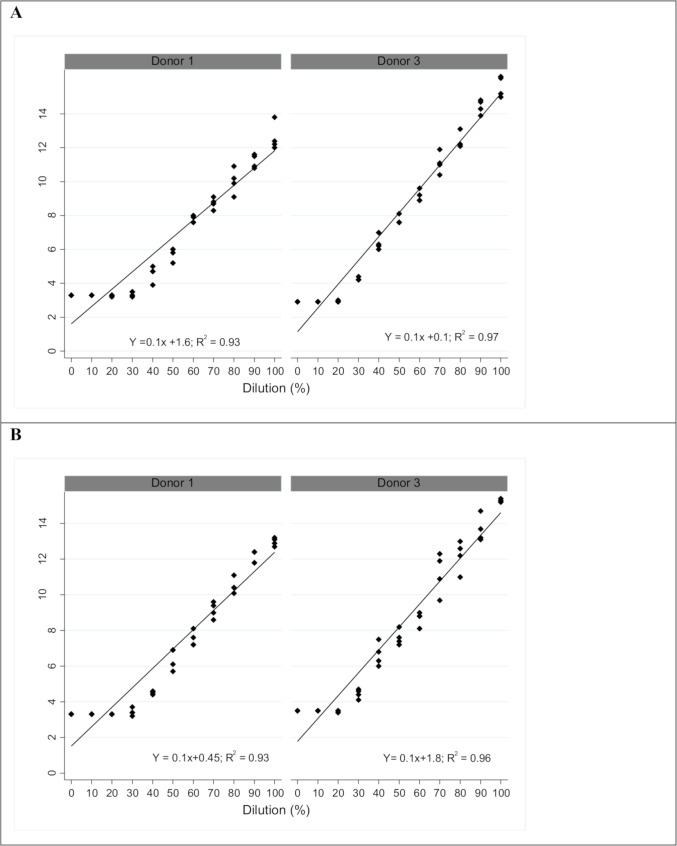
An assessment of TrueHb linearity at (A) 30°C and (B) 37°C.

Three linearity panels at 21°C, one for each donor, are shown in Fig 2. The individual TrueHb data points results are plotted; the average TrueHb linear fit line and R^2^ values are given. At room temperature (21°C), the TrueHb follows a linear relationship (0.98, 0.96 and 0.97). However, the TrueHb did not generate accurate hemoglobin readings below 4.0 g/dl at 21°C. Fig 3 shows the linearity panels at 30°C and 37°C for two of the same donors; TrueHb follows a linear relationship (R^2^ > 0.90) but begins to underestimate the hemoglobin concentration below 7.0 g/dl.

### Usability study

Twenty health workers were recruited and participated in the usability evaluation, including: six midwives, twelve nurses, one physician assistant, and one lab assistant. On average, participants had three years of health care work experience. When we asked about methods used for anemia screening, participants reported experience using the WHO color scale, clinical assessment, and the URIT 12 hemoglobin measurement tool (Shanghai SUCE Medical Technology Development Co., Ltd./Guangxi, China).

**Fig 4:**
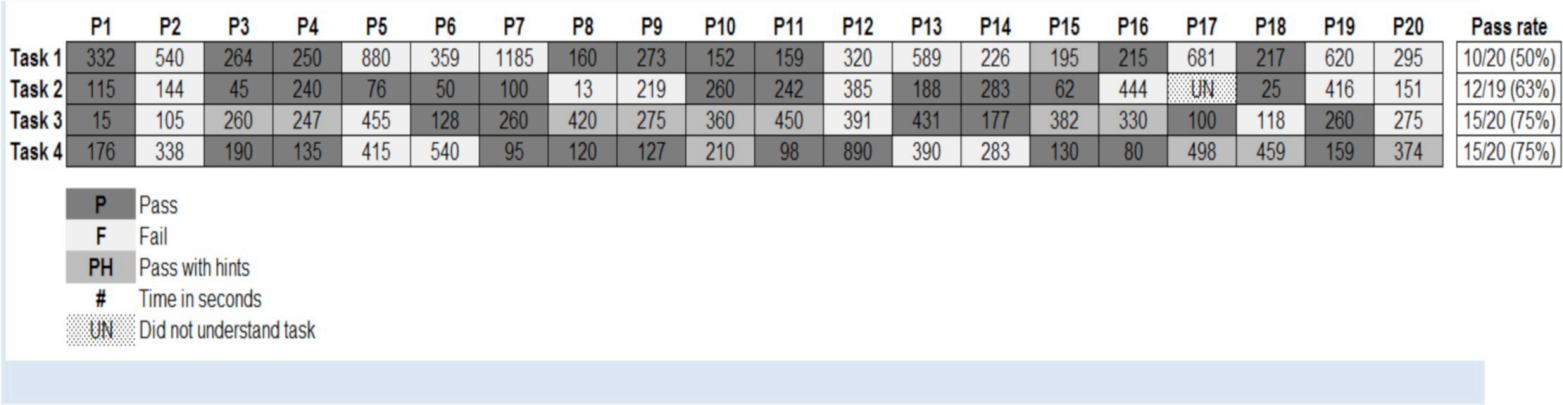
Summary of task success rates by participant.

Overall task completion rates ranged from 50–75%. There was no relationship between task success and years of experience or education. Fig 4 provides a visual depiction of pass and fail rates. Each column in this grid represents a participant and each row is a task. A dark grey box indicates the user passed that task, white that he/she failed, and light grey that he/she was able to pass but needed some hints from the facilitators.

Two catastrophic issues were identified: the use of insufficient blood volume (three occurrences) and premature removal of the strip from the device (five occurrences). Both of these errors lead to inaccurate results and the user was unaware. Instead, an inaccurate low result is given. Additionally, two critical issues were identified: placement of the strip and blood. Participants inserted the strip incorrectly a total of 18 times across 40 Hb measurement attempts. The most common error was not inserting the strip far enough into the device. When this happened, it often led to blood placement errors. Other less common errors were inserting the strip backwards or upside down. In total, there were five instances of the blood being added to an incorrect area of the strip. In four of those instances, blood placement errors coincided with the strip being incorrectly inserted into the device.

**Fig 5:**
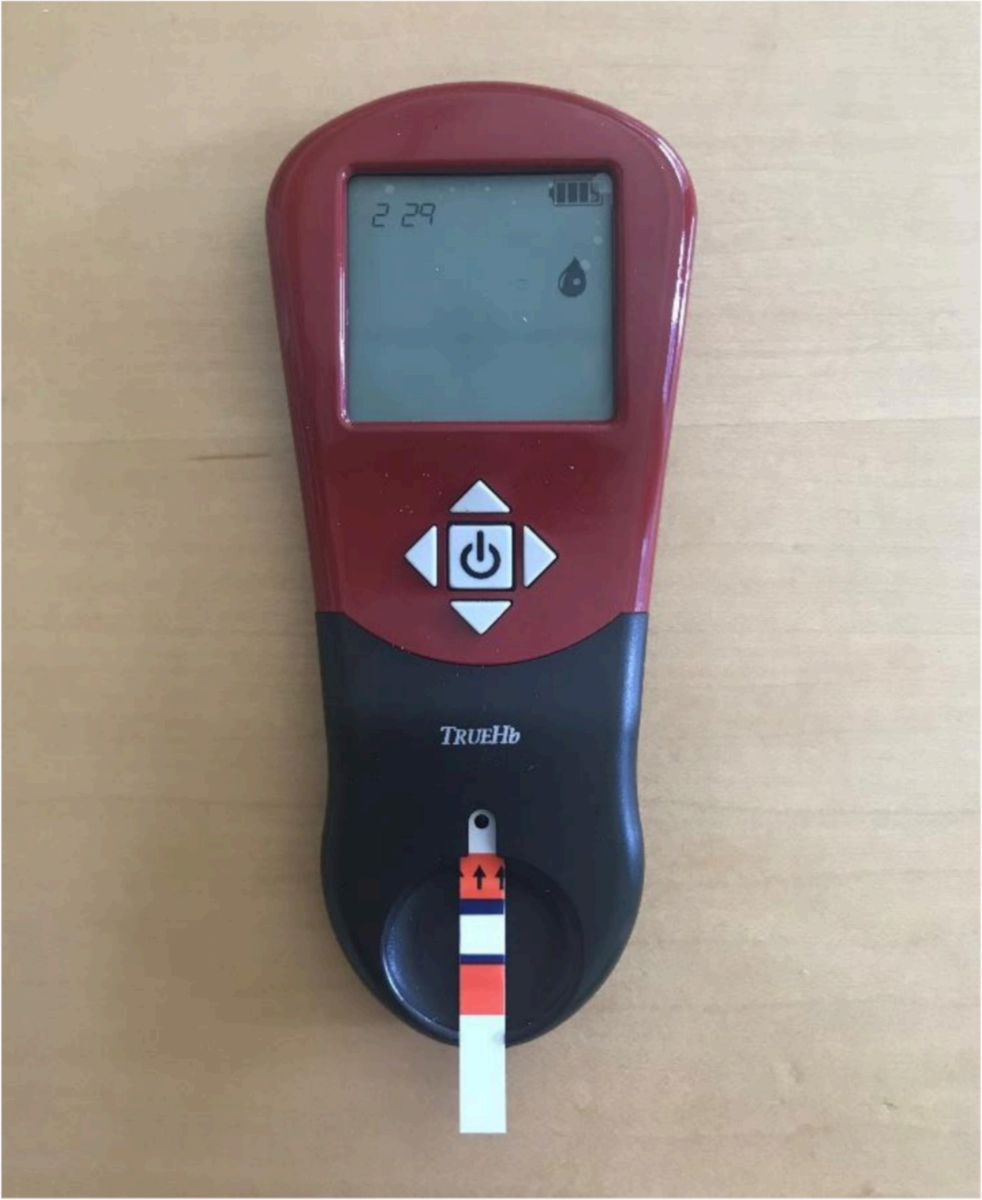
An image of the TrueHb device.

A major issue that challenged participants was the process of entering and accepting the batch code. Each container of TrueHb strips has a unique batch code. The user must adjust the batch code to match the code on the strip container and press accept before taking a measurement. Using the left and right arrows to navigate between digits of the code and the up and down arrows to change the numbers was not intuitive for the majority of participants (Fig 5).

Accepting the batch code by pressing the middle button was also challenging. A likely cause of this confusion was the dual purpose of the middle button. This button is both the “enter” button and the power on and off button. Participants seemed hesitant to push this button for fear of powering down the device while in use.

The primary driver of these failure modes was likely the inaccurate and inadequate instructions for use (IFU) included in the test kit. There were inconsistencies with the features of the device and those described in the instructions. When asked about their impressions of the instructions, some participants commented on the density of the text and the length, suggesting that more images could be used to enhance clarity and ease of use. While these issues are considered major, the inaccuracies and inadequacies of the instructions can be readily addressed in future versions.

Regardless of these challenges, ease of use ratings were consistently high across tasks, with a majority of participants reporting ratings of 4 or 5 on a Likert scale for each task.

## Discussion

With some modifications, both to the product and to the instructions for use, the TrueHb may be suitable for use in ANC settings and help fill the diagnostic gap for anemia screening during pregnancy. Additional use cases for the tool’s utility in anemia screening could also be explored including adolescent and child screening, as well as research. The capillary Hb readings generated by the TrueHb and HemoCue 201+ are not highly correlated; however, the majority (80%) of the TrueHb results fell within 1.0 g/dl of the HemoCue® 201+. Further studies are needed to examine the accuracy of the TrueHb hemometer in comparison to both the HemoCue 201+ and an automated hematology analyzer (gold standard) using a larger sample size in a setting of intended use where the prevalence of anemia is at least 40% among pregnant women. In order to have accurate measures of sensitivity and specificity at clinical thresholds for anemia and severe anemia, it is necessary to include sample participants with hemoglobin concentrations at the lower end of the hemoglobin range, below 11.0g/dl and 7.0g/dl. Further research with capillary samples is needed and factors including temperature, altitude, patient hydration level, and trimester should be tested as they can each play a role in the performance of hemoglobin POC devices.

The venous study provided performance data across a dynamic range of hemoglobin values at varying temperatures and humidities. As a comparison, the HemoCue 201+ device accurately reads hemoglobin concentrations between 0 and 25.6g/dl for temperatures of 15 to 30°C [25]. Our laboratory evaluation of the TrueHB found that up to 30°C and 50% humidity, the TrueHb had acceptable repeatability (<6%). However, considering many low-income countries experience temperatures up to 35°C and 40°C, further precision testing should be conducted to determine the need for product adjustments. The linearity assessments suggested that the TrueHb may underestimate hemoglobin values in the lower range, below 7.0 g/dl, which is a critical threshold for severe anemia. This could lead to more women being falsely categorized as having severe anemia, which would in turn necessitate referral for additional evaluation. This would limit the utility and cost-effectiveness of the tool in ANC settings. Moreover, as we found the TrueHb cannot reliably measure hemoglobin levels below 4.0g/dl, we recommend that when hemoglobin values below 4.0g/dl are detected, the machine report error codes, rather than a value, in order to prompt alternate testing.

The usability study identified potential failure modes that should be addressed before the test is introduced in ANC settings at scale. Two key principles of good interaction design are that the user should feel empowered, and the designer should make it easy for users to diagnose and recover from errors. We have identified two root causes of the observed TrueHb usability issues: (1) the instructions and (2) the device feedback. These issues reduce user effectiveness, efficiency, and satisfaction and should be the focus of product improvement efforts. The instructions should be revised to remove inaccuracies and reorganized to better reflect the user workflow.

Device feedback could be improved with the addition of error codes on insufficient blood volume, out of range values, and premature removal of the test strip. The batch code entry process could be clarified with improved text and illustrations in the instructions or a device redesign where strips are automatically read and assigned a batch code. The power button and enter button should be made distinct or clarified with onscreen prompting. Strip and blood placement errors could be reduced by adding an instruction that links the green light emitted by the device to blood placement, or the addition of arrows to the strip that highlight the stopping point for insertion with clarifications in the instructions. Improved video training resources on test use would also aid in improved usability.

### Limitations

This study has several limitations. First, the capillary study did not include a venous blood draw to use in an automated hematology analyzer as the gold standard or multiple finger sticks to enable multiple test replicates. A second limitation of the capillary study was that the population sampled at PATH were mostly healthy, non-anemic adults in a high-income country setting. The sample set contained only two cases of anemia (<12.0 g/dl; non-pregnant women; <13.0g/dl adult males), and thus we cannot report on the sensitivity and specificity of the TrueHb tool for anemia detection.

For the venous study and the usability study, it would have been helpful if this study had tested the both HemoCue201+ and TrueHB for direct comparisons in terms of precision, reliability, and user preference. During the usability study, some confounding factors may have influenced the results. To reduce the impact of a decaying attention span among participants, the investigators attempted to contain usability testing sessions to one-hour. However, in some cases interviews exceeded this length due to participant-specific factors, such as speed and meticulousness reading the instructions and tenacity to successfully complete tasks. This may have impacted answer quality and comparability. Considering that the study was observational in nature, it is possible that the presence of the investigators influenced the health practitioner’s ease of use ratings reported. Lastly, participants will traditionally perform better on tasks performed later in the data collection session and the investigators did not control for this issue by randomizing the order of tasks to be completed.

## Conclusions

ANC clinics in LMIC need a POC hemoglobinometer tool that can accurately detect anemia and severe anemia in pregnant women. Less expensive tools are more accessible in LMIC settings and the TrueHB costs approximately $35USD while the HemoCue 201+ costs $320USD. Access to such a tool will help clinicians’ better track patient progress and choose treatment options and may also motivate pregnant women to adhere to IFA supplement regimens. In the absence of better alternatives that are currently available, the TrueHb may be sufficient to fill this diagnostic gap, but iterations in product design and testing are needed. Further data using a representative and sufficient sample set is critical to understand test performance in capillary samples. Additionally, operation and cost-effectiveness studies are needed to understand the implications of under- or overestimating hemoglobin levels, particularly near the threshold for severe anemia.
